# Integrated GC–MS and ATR–FTIR analysis of airborne microplastics captured by spider webs

**DOI:** 10.1038/s41598-026-57675-z

**Published:** 2026-06-11

**Authors:** Elżbieta Szostak, Magdalena Wróbel, Beata Kultys, Joanna Kamińska, Piotr Duda, Justyna Rybak

**Affiliations:** 1 Faculty of Chemistry, Jagiellonian University, Gronostajowa 2, 30- 387 Kraków, Poland; 2 Faculty of Environmental Engineering, Wrocław University of Science and Technology, Wybrzeże Wyspiańskiego 27, 50370 Wrocław, Poland; 3 Department of Applied Mathematics, Wrocław University of Environmental and Life Sciences, Norwida 25, 50-375 Wrocław, Poland; 4 Faculty of Mechanical Engineering, Cracow University of Technology, Warszawska 24, 31-155, Kraków, Poland

**Keywords:** Microplastics, Spider webs, Environmental monitoring, Urban pollution, Poland, Chemistry, Environmental sciences, Materials science

## Abstract

Our study evaluated airborne microplastics (MPs) captured by spider webs (Agelenidae) in an urban environment (Wrocław, Poland). GC–MS was used to characterize extractable organic compounds linked to MPs, while ATR–FTIR spectroscopy enabled detection of polymer-related signals in solid residues after solvent extraction. All samples contained compounds associated with polymeric materials, including rubber- and silicone-related components, with rubber signals detected at all sites. No significant differences were observed between central and suburban locations, indicating a relatively uniform distribution of airborne MPs. ATR–FTIR results further suggested the occurrence of tire-derived materials in all samples, along with polyamides (Nylon 6; 40%), polypropylene-related materials (including Epcar 5875; ~20%), polyvinyl alcohol (20%), and polyethylene (LDPE; 13%), expressed as frequency of occurrence across samples. Overall, the results demonstrate that spider webs can serve as accessible passive samplers, providing a qualitative and integrative representation of airborne microplastic contamination in urban environments.

**Supplementary Information:**

The online version contains supplementary material available at 10.1038/s41598-026-57675-z.

## Introduction

### Microplastics as emerging atmospheric contaminants

Since the 1950 s, polymeric materials have been an integral part of human life in many spheres, from simple everyday tasks to various technological systems^1^. Their use is typically motivated by financial considerations and, in certain situations, they represent the only viable option. As plastic production continues to grow rapidly, their waste also increases. A significant part of this waste ends up in landfills, although a large share is recycled for energy purposes (via combustion), chemical recovery (such as pyrolysis, depolymerization), or mechanical processes (like segregation and reprocessing)^2^. Over time, plastic waste degrades, posing a major threat to ecosystem^1^. One particularly pressing concern is the presence of microplastic polymer particles, commonly known as microplastics (MPs) in the environment. The issue extends far beyond water pollution. Although MPs were first identified in marine environments, they can also be found in freshwater systems^3^, terrestrial ecosystems^4^, and the atmosphere^5^. While research on MPs has largely focused on marine ecosystems, terrestrial and atmospheric MP contamination has only recently received attention. Given that, airborne MPs can enter the human body directly and constantly through inhalation, awareness of this exposure route is growing^6^. Airborne MPs behave similarly to other airborne pollutants and have been detected both indoors and outdoors with significantly higher concentrations indoors^7^. However, there is still limited knowledge about the presence, sources, and behaviour of airborne MPs, particularly in urban and industrial areas, where human exposure is highest^8^.

Microplastics are now ubiquitous- even present in the human body. They are inhaled and ingested^9^. MPs pose potentials risks to living organisms and can further break down intonanosized particles, which may be even more harmful^9^. Studies have shown that MPs can attach to surfaces and act as carriers for other pollutants^10^. For these reasons, it is essential to continuously monitor the occurrence of MPs in the environment and evaluate their impact on the e ecosystems, and human health.

### Spider webs as passive biosamplers

Spiders play an important role in ecosystems and can serve as bioindicators due to their widespread presence, including highly polluted areas^11^. The majority of studies have focused on webs of spiders from Agelenidae family. These spiders construct a flat sheet of non-sticky web with a funnel-shaped retreat to one side at the center. Numerous studies have demonstrated that such webs are highly effective for environmental monitoring as their dense, extensive web structure efficiently traps airborne pollutants.

To date, the identification of MPs on spider webs has been conducted in only a few countries^5,12^ and no such studies has been performed in Poland, creating an important regional knowledge gap. As spider webs show an excellent capacity for tracking the urban aeolian particle load, our research aimed to assess the presence of MPs in urban air by analyzing particles trapped on spider webs.

### The need for integrated analytical approaches (GC–MS + ATR-FTIR)

Within this work, we proposed to combine GC-MS and FTIR techniques to enhance MP contamination assessment of spider web samples by providing their comprehensive identification. This combined approach is rarely applied but offers clear methodological advantages, providing a more complete picture of MP contamination.

- The high sensitivity of the GC-MS method allows detection of contamination even at the nano level.
- FTIR spectroscopy provides complementary information about the functional groups and overall molecular structure of the larger MPs present in spider webs.

By combining these techniques, we can gain a more comprehensive understanding of MP contamination in environmental samples.

FTIR spectroscopy has become a key tool for identifying and quantifying MPs in various environments. Veerasingam et al.^13^ highlighted the rapid development of FTIR techniques and suggested their potential application to spider web analysis, which could allow for the identification of MP species captured by spiders during web construction. The authors also emphasized the usefulness of µFTIR imaging for visualizing the distribution of MPs in various environmental matrices, potentially including spider webs. The ability of FTIR spectroscopy to identify multiple polymer types suggests that this method could be a key tool in studying MPs trapped on spider webs. It could also aid in tracking the environmental fate of MPs and understanding how spider webs function as passive traps for airborne particles^13^. Although FTIR spectroscopy is well established in MP analysis, gas chromatography-mass spectrometry (GC–MS), particularly in its pyrolytic version (Py-GC/MS), is gaining increasing importance for characterizing complex polymer mixtures. Ribeiro et al.^14^ demonstrated the successful use of Py-GC/MS for detecting MPs in marine organisms, suggesting that similar procedures could be adapted to spider webs. Dissolution of MPs in a suitable solvent may be crucial in such analyses. Fuller and Gautam^15^ described a pressurized fluid extraction (PFE) method for determining MPs in environmental samples, which can be adapted by using nonpolar solvents such as hexane to increase the solubility of hydrophobic polymers. This suggests that hexane may improve the recovery of MPs prior to GC–MS analysis, but its application to spider-web samples remains unexplored.

Zhang et al.^16^ discussed the challenges of analyzing MPs in soil matrices, pointing out that the smallest particles often require innovative extraction methods. The use of hexane can simplify the process and increase recovery efficiency. Similarly, Dehaut et al.^17^ developed a reference protocol for the extraction and characterization of MPs in marine organisms, emphasizing the importance of reliable and standardized procedures. Their study suggests that the inclusion of solvents such as hexane can increase the efficiency and accuracy of analyses. Ivleva^18^ highlighted the complexity of MP analysis due to their diverse chemical and physical properties. Therefore, advanced techniques such as GC–MS are particularly useful for analyzing MPs dissolved in solvents such as hexane. Hendrickson et al.^19^ indicated that standard analytical protocols can incorporate the use of hexane as a solvent in combination with GC–MS, especially when analyzing drinking water. Primpke et al.^20^ critically compared Py-GC/MS and FTIR hyperspectral imaging, emphasizing their complementarity. Using FTIR for the initial identification of polymers and Py-GC/MS for quantitative confirmation of results can provide a comprehensive characterization of MPs accumulated in spider webs.

Despite constant progress in the development of MP analysis methods, significant knowledge gaps remain regarding both methodological aspects and the interactions between MPs and spider webs. Future research should focus on integrated methods combining FTIR and GC–MS to obtain more accurate quantitative and qualitative data. The use of hexane as a solvent prior to GC–MS analysis remains underexplored, and therefore, studies are needed to optimize its effectiveness on different types of MPs with differing hydrophobic properties. Comparative studies on the effectiveness of hexane and other solvents in the dissolution and extraction of MPs are also worthwhile.

### Knowledge gaps and the need for field studies

Despite constant progress, significant knowledge gaps remain regarding:

- analytical methods for MPs in complex biological matrices like webs,
- interactions between MPs and spider silk,
- the role of spiders in accumulating airborne MPs under real environmental conditions.

To date, research on the presence of MP in the atmosphere has focused almost exclusively on active collection methods, such as high-flow filters and aerosol vacuum cleaners^21,22^. While valuable they do not allow long-term deposition integration and require power and maintenance. Simple passive methods such spider webs could fill this methodological gap. Furthermore, little is known about how environmental conditions affect particle capture efficiency, the effects of MP accumulation on spiders, or how webs accumulate MPs over time. Long-term monitoring studies will allow for the assessment of temporal changes in MP accumulation in spider webs and provide data on the persistence and degradation of these pollutants in the atmosphere.

Moreover, most existing studies focus solely on identifying polymer types, neglecting additive compounds (plasticizers, antioxidants, silicones) that may coexist with MPs and influence their toxicity^23^. To date, no studies have been conducted combining solvent extract analysis (GC–MS) with solid-state polymer identification (ATR–FTIR) for spider webs.

### Aim and objectives of the study

Our study fills this gap by proposing an integrated analytical approach (GC–MS + ATR–FTIR) and assessing the suitability of Agelenidae spider webs as bioindicators of MP pollution in urban environments. This is the first study of its kind in Poland and one of the few in Central and Eastern Europe^5,12^.

This preliminary study had two main objectives:

1. To generate a profile of extractable and leachable substances from spider web samples, and assess their potential correlation with the presence of microplastics.
2. To assess the possibility of using the ATR-FTIR spectroscopy to detect and identify MPs in residues after solvent evaporation.

We hypothesized that spider webs collected from various urban locations - with differing traffic intensity therefore, pollution levels - would show variation in the composition and types of pollutants adsorbed on the webs. Additionally, we assumed that comparing two analytical methods would provide more comprehensive information about the types of MPs present on the webs.

## Materials and methods

### Chemicals and materials

n-hexane (HPLC grade) was purchased from Chempur (Piekary Slaskie, Poland). Hydrogen peroxide (30% w/w in H_2_O), ferrous sulfate heptahydrate (FeSO_4_$\:\bullet\:$7H_2_O) and sulfuric acid (H_2_SO_4_, 98%) were obtained from Merck (Darmstadt, Germany). A certified reference mixture of C9 – C33 saturated alkanes (1000 µg/mL in hexane) was also obtained from Merck.

### Spider webs location and collection

In this study, we did not conduct parallel URD collection using active measurement methods, such as particulate matter collectors (e.g., PM10/PM2.5 devices, impactors, aspirators, or gravimetric collectors). The study was designed to evaluate spider webs as passive samplers of airborne MPs. This method allows for low-cost research without the use of specialized aerosol collection devices. Two species from the Agelenidae family *Agelena labyrinthica* (Clerck 1757) and *Tegenaria ferruginea* (Panzer) were employed in the study in Wrocław, Poland. The web is typical of all spiders in the Agelenidae, also known as funnel spiders, which is made up of a sheet web with a tubular retreat or funnel where the spider waits for its prey. All of the silk used to make the web is dry. This kind of web’s non-stickiness makes it simple to capture contaminants, especially micropollutants, due to its compact, rigid, and dense structure. Agelenid spiders have already been shown to be very useful for air pollution biomonitoring^24^. Bushes and hedges are among the vegetation in which *Agelena labyrinthica* thrives. Both environments are also home to *Tegenaria ferruginea*, which can be found in buildings, woodlands, and close to the ground^25^.

We employed the naturally occurring webs of these two spider species. Only newly formed webs were sampled following prior removal of pre-existing webs to ensure controlled exposure time. Samples were collected at 15 locations, representing areas of varying traffic intensity. Each sample consisted of pooled material from at least three webs collected at a single site. Pooling was applied to obtain sufficient material mass for chemical analysis; therefore, samples represent composite site-specific matrices rather than biological replicates.

For studies in situ sampling was applied, and the webs were collected using a glass stick and kept in a glass vial, webs were collected at similar heights (close to ground level, approximately 0.5–1 m). The sampling duration was eight weeks, from the beginning of September until the end of December 2022 (autumn to early winter) in the city of Wrocław (Poland). The selected 8-week exposure period was deliberately chosen as a compromise between practical feasibility and environmental relevance. This time frame covers the transition phase from early autumn to early winter in Eastern Europe, which includes changes in weather conditions, leaf fall and the beginning of the heating season – factors that can affect the composition of airborne particulate matter. Although this period does not cover the full seasonal cycle, our main goal was to assess the feasibility of spider webs as passive airborne particulate matter samplers in real urban conditions. All samples were handled using glass equipment to minimize contamination from synthetic polymers.

**Fig. 1:**
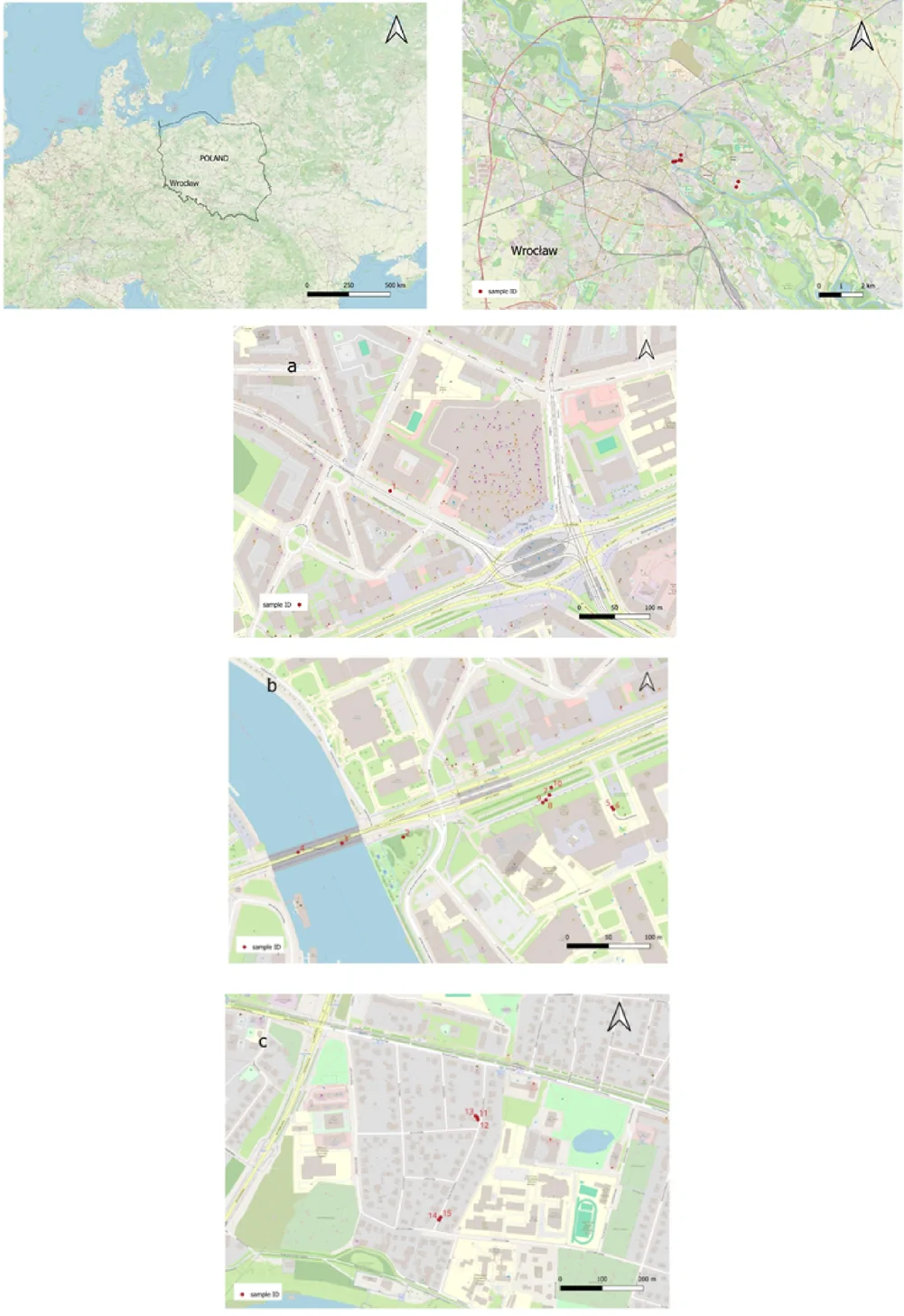
Sampling sites’ map (a – site 1, b – sites 2–10, c – sites 11–15). Urban and suburban sampling sites differed in surrounding traffic intensity, vegetation cover and building density (see Table S1 in the Supplementary Materials). Maps were created by the authors using QGIS Desktop v. 3.xx (https://qgis.org) with OpenStreetMap basemap data (© OpenStreetMap contributors, https://www.openstreetmap.org)^26^.

Average weather at this time included moderate temperatures, light to moderate winds, and sporadic rain. The detailed description of sampling sites is presented in Supplementary materials table S1. Figure 1 shows the localization of sampling points.

### Sample preparation

Samples were pre-dried at 90 °C for 72 h and weighed using an analytical balance (Radwag AS R1 PLUS; Table S2). The drying step was applied to remove moisture and standardize sample mass prior to digestion. Each sample was transferred to a 150 mL glass beaker, and 20 mL of 30% H₂O₂ was added. Samples were incubated for 16 h at room temperature. Subsequently, 10 mL of 0.05 M FeSO₄ solution acidified to pH 3 with H₂SO₄ was added to initiate Fenton-like digestion^27^. The catalyst was added incrementally (2 mL every ~ 10 min), and the reaction temperature was maintained below 60 °C. After completion of the reaction (4–6 h), samples were diluted with ultrapure water and filtered to separate and concentrate solid particles, including MPs, from the digested matrix prior to solvent extraction. The digestion procedure was repeated to improve removal of organic matter. In the final step, 2 mL of n-hexane was added to each sample to extract solvent-soluble organic compounds associated with MP particles.

Control samples (*n* = 6) consisted of spider webs produced under laboratory conditions and stored in sealed containers without environmental exposure. These controls underwent identical preparation.

Recovery experiments were conducted using commercially available reference polymers subjected to the full sample preparation procedure. Recovery efficiencies ranged between approximately 70% and 90%.

### GC-MS analysis

Hexane extraction was applied to isolate solvent-extractable organic compounds associated with MP particles, including low-molecular-weight degradation products and polymer additives. The use of non-polar solvents such as hexane for the extraction of polymer-related compounds and additives has been previously reported in studies on plastic degradation and chemical characterization of polymer materials^28–30^. Extracts were subjected to mechanical shaking (IKA HS 260 basic) for five days (6 h per day, 180 rpm, ambient temperature) to enhance extraction efficiency.

Aliquots (1 µL) of the supernatant were injected into a Shimadzu GC-2030 gas chromatograph coupled with a triple quadrupole mass spectrometer (MS-TQ8040 NX) equipped with an AOC-600 autosampler. Data acquisition and processing were performed using LabSolutions software (version 4.53). Chromatographic separation was carried out on an SH-5MS capillary column (30 m × 0.25 mm × 0.25 μm). Helium was used as the carrier gas at a constant flow rate of 1.2 mL/min. The GC oven temperature program was as follows: initial temperature 50 °C, ramped to 320 °C at 15 °C/min, and held for 15 min (total run time: 34 min). The injector temperature was set to 280 °C and operated in splitless mode. Mass spectrometric detection was performed using electron ionization (70 eV) in SCAN mode over the m/z range 30–400. The ion source and quadrupole temperatures were maintained at 200 °C and 250 °C, respectively.

Compound identification was based on comparison with analytical standards, spectral matching using the NIST 2014 Mass Spectral Library, and consistency of fragmentation patterns. Only compounds with high spectral match quality were considered. To verify system performance and support compound identification, a standard mixture of saturated hydrocarbons (C9–C33) was analyzed under identical conditions. Limits of detection (LOD) were determined using a signal-to-noise ratio (S/*N* = 3). For selected hydrocarbons (C14–C20), LOD values ranged from 3 to 5 pg, corresponding to 3–5 ng per sample.

GC–MS analysis does not allow direct identification of intact polymer particles. Instead, it provides information on extractable organic compounds associated with MPs, such as additives, oligomers, and degradation products.

Therefore, assignment of detected compounds to polymer-associated material groups was based on literature reports describing characteristic additives, oligomers, degradation products, and co-occurring compounds linked to particular polymer families (see Table S3 and corresponding references). Individual compounds were not considered unique diagnostic markers, as some compounds may occur in multiple polymer types or originate from mixed anthropogenic sources. To support interpretation, relationships among detected compounds were additionally evaluated using correlation analysis (31 × 31 matrix). Groups of compounds showing consistent co-occurrence patterns across samples were interpreted as indicative of polymer-associated material groups rather than direct identification of specific polymers.

### ATR – FTIR

Infrared spectra were recorded in the 4000–400 cm⁻¹ range using a Bruker VERTEX 70v spectrometer (Ettlingen, Germany) equipped with a diamond ATR accessory (Bruker A225/Q-DLST). Spectra were collected with 64 scans at a resolution of 4 cm⁻¹. Background spectra were recorded prior to each measurement. Prior to analysis, samples were homogenized by shaking for 30 min. Aliquots of the extract were deposited onto the ATR crystal in a dropwise manner, allowing complete solvent evaporation between successive additions. ATR–FTIR analysis was performed on residues remaining after hexane evaporation and therefore represents the non-extractable fraction of particles. Polymer identification was carried out by comparison with reference spectra using the OPUS 8.5 software (Bruker Optik GmbH). Spectral preprocessing included baseline correction, smoothing, and normalization. Polymer assignment was based on characteristic absorption bands and library matching. Due to methodological limitations, ATR–FTIR analysis is biased toward larger particles (typically > 300 μm), while smaller or transparent particles may remain undetected.

### Quality assurance, contamination control, and method validation

Blank samples consisting of all reagents processed without sample material (*n* = 3) showed negligible signals corresponding to MP-related compounds in both GC–MS and ATR–FTIR analyses. No MP-related signals were also detected in control spider’s web samples (*n* = 6), confirming that contamination during handling and preparation was negligible.

The applied methodology combines solvent extraction (GC–MS) and ATR–FTIR spectroscopy, targeting different fractions of MP-associated material. GC–MS analysis reflects extractable organic compounds (e.g., additives and degradation products), which are not uniquely specific to individual polymer types. Therefore, compound assignment should be interpreted as indicative rather than definitive. The proposed approach should therefore be interpreted as a qualitative or semi-qualitative assessment of polymer-associated material signatures rather than direct polymer identification based solely on GC–MS data. ATR–FTIR analysis was performed on residues after solvent evaporation and is therefore biased toward larger particles (typically > 300 μm) and non-soluble fractions. Smaller or transparent particles may remain undetected.The method does not allow direct quantification of particle number or mass, and results are therefore interpreted in a qualitative or semi-quantitative manner.

The complete analytical workflow applied in this study is summarized in Figure S1 in the Supplementary Materials.

## Results and discussion

### GC-MS analysis

#### Methodological considerations

Interpretation of GC–MS results in this study should consider the limitations associated with the analysis of complex environmental matrices such as spider webs. Hurley et al^31^. emphasize that using Fenton’s reagent improves the removal efficiency of organic matter, increasing extraction efficiency while minimizing degradation of MP particles. Other factors that may interfere with the detection of MPs leading to inconsistent detection limits and sensitivity are the variability in size, shape, and types of polymers^18^. The detected compounds represent solvent-extractable organic fractions associated with microplastics, including additives and degradation products, rather than intact polymer structures. Therefore, assignment of compounds to specific polymer types is based on literature-reported marker compounds and should be interpreted as indicative, particularly in the case of mixed or degraded polymers^32,33^. To address these limitations, ATR–FTIR analysis was applied as a complementary technique, enabling identification of solid polymer residues and providing additional support for the presence of microplastic particles.

#### Composition of the extractable fraction

Figure S2 shows the chromatograms obtained by GC-MS for all analyzed samples, including blank solvent. The comparison of the extractables profiles with MS analysis revealed that all extracts contain compounds that may be associated with materials such as plastic, rubber, and/or silicone crumbs. These compounds were assigned to a specific polymer type based on literature- reported marker compounds, and, where possible, to their functional roles as additives or degradation products.

The relationships in the occurrence of the identified compounds in the studied samples were described using the correlation coefficient matrix (Fig. S3). Groups of compounds that occur simultaneously in the samples are marked.

Semi-quantitative conclusions were made through comparative evaluation of the GC-MS peaks area recorded for individual spider web samples. Analysis of the data collected in Table S4 and shown in Fig. S3 allows us to conclude that all collected webs contained compounds associated with rubber -derived materials, with the highest amount of these compounds detected in sample 5 and the lowest in sample 8. Signals related to the presence of rubber particles in spider webs are correlated with each other and come either from compounds directly bound to the rubber composition - signals 4 and 6–11^34,35^ from chemical additives used during its production such as antioxidants - signal 14^36^ or lubricants - signal 26^37^. The next group consisted of correlated signals 15, 17, 19, 22, 25, and 28 associated with polyethylene fragments (PE) in the samples. Signal 22, directly related to the polymer backbone fragments was characterized by the largest peak area, and was therefore considered the most representative of the actual PE content in the spider web samples. The remaining signals were associated with the presence of volatile organic compounds^38^, or slip agents^38–42^, and surfactant/lubricants^39,43^ used as chemical additives for PE. The highest relative abundance of PE – associated compounds was noted in sample 14, while the lowest in sample 10.

All extracts contained cyclic and linear siloxanes (signals 5, 12, and 18), which as indicated in the literature may originate from silicone- based materials intended for food contact^44^ but also from consumer products such as cosmetics personal care products and building materials^45^. Studies performed by Okpashi et al^46^. revealed that siloxanes derivatives may also leached from polyethylene sachets during their exposure to ultraviolet radiation. Silicones are commonly used as ingredients in lubricants and polymers. Due to their widespread use they may occur in air and outdoor dust^47^. The highest siloxane concentrations were observed in sample 13 and the lowest in samples 8, 11 and 12. Signals 1 and 16 indicate the presence of branched and unbranched alkanes, which have been reported in association with polypropylene (PP) particles^42,48–50^. Their presence may be related to degradation of the polymers due to aging and/or recycling processes^51^. PP- related markers were detected in all tested samples, with the highest relative abundance in samples 12 and 13 and the lowest in sample 8. Signals 2 and 21 were registered for all tested samples and were associated with compounds inked to polyacrylonitrile (PAN) and polyamide (PA), respectively^36,52,53^.

These polymers are used in the textile industry and most likely originate from fragments of synthetic fibers used in the production of fabrics. The highest relative abundance of PA and PAN – associated compounds was noted in sample 8, while the lowest in samples 6, 11, and 12. Signal 3, was the largest for sample 3 but was not recorded for samples 7 and 11. According to the literature data^36^, it may be associated with polyurethane (PUR) – related compounds in the tested sample. Relatively high intensities were shown by signals 13 and 24 - sample 1, signal 23 - samples 1, 5 and 11, and signals 29 and 30 - sample 12. These signals may be associated with compounds reported for polyethylene terephthalate (PET)^54–58^, polystyrene (PS)^41,59^ and polyvinyl chloride (PVC)^41^, respectively. It is noteworthy that most of these signals are associated with the phthalate esters used as plasticizers. The presence of such compounds in the environment may interfere with endocrine functions, which is why phthalate esters are classified as endocrine-disrupting chemicals (EDCs)^60^. Similarly, very intensive signals were registered for tetrapentacontane and 1,4-Epoxynaphthalene-1(2 H)-methanol, 4,5,7-tris(1,1-dimethylethyl)−3,4-dihydro-, which may be related to degradation products of plastic materials^61–63^. The extent of such degradation depends on polymer type, environmental conditions, and biological activity, and may therefore vary between sampling sites.

To summarize, the complex and heterogeneous nature of spider webs as environmental matrices makes direct identification and quantification of individual MPs particles inherently challenging. However, this characteristic also reflects real environmental conditions, where airborne particles accumulate over time and originate from multiple sources.

#### Spatial patterns and lack of urban gradient

To sum up, our results do not reveal statistically significant differences between central and suburban locations in terms of the presence and types of MPs. This finding can be attributed to several environmental and urban factors.

First, despite differences in traffic density, Wrocław was identified as one of the most congested cities in the Europe among cities with a population of less than 800,000, with heavy vehicle traffic even in areas considered suburban (e.g^64^). According to the TomTom Traffic Index, drivers in Wrocław experience one of the longest traffic delays, suggesting that even “suburban” locations may experience significant MP impacts from traffic sources (https://www.tomtom.com/traffic-index/ranking/).

Second, our analysis confirmed the presence of compounds associated with tire wear (rubber-derived materials) in all collected samples, regardless of location. The uniform distribution of such compounds suggests that dispersion and resuspension processes in the atmosphere may play a major role in the spread of MP across urban gradients.

Furthermore, several factors may also explain our findings, despite previous reports of traffic-related gradients in MP pollution (e.g^12^). :

- urban structure and wind patterns: Wrocław is characterized by dense urban morphology and frequent wind corridors along the Oder River., which may enhance the atmospheric mixing and dispersion of MPs, reducing location-depending differences^65^.
- consistency of sampling height: webs were collected at similar heights (0.5–1 m above ground), which minimizes vertical variability. In contrast, stronger gradients are often observed at higher altitudes or near emission sources^66^.
- homogenized background pollution: urban environments may reach a relatively uniform background levelz of MPs due to atmospheric mixing, especially when traffic density does not differ drastically between locations—as is the case in Wrocław, where traffic is heavy even in peripheral areas^7^.
- dominance of tire-related particles: the widespread occurrence of rubber-associated compounds across all sites suggests that resuspension and regional atmospheric transport may be more important than local emission sources^67^.

Finally, the lack of a noticeable mitigating effect from vegetation may be due to the sampling period (autumn to early winter) when leaf cover was reduced, limiting the barrier function of green infrastructure. Although, urban greenery is typically viewed as a barrier limiting the deposition of airborne particles, its role in MP cycling may be more complex. MPs accumulating on leaves, bark, or cobwebs in green belts can be re-released and transferred to the soil, especially during rainfall or leaf fall. Consequently, greenery may act not only as a sink but also as a secondary source of MPs to the soil, particularly during the leafless period, when the particle retention capacity is limited^68^. Such seasonal effects may explain the local increases in MP counts observed near green belts and suggest the need to consider temporal variability when interpreting results.

### ATR-FIR analysis

ATR-FTIR can detect plastic particles at a resolution of 10 μm, however, its effectiveness depends on particle size and morphology, as it easily detects thick microplastics, while thin and small ones may remain undetected. Therefore, it is primarily applied to analyze larger particles (> 300 μm).

**Fig. 2:**
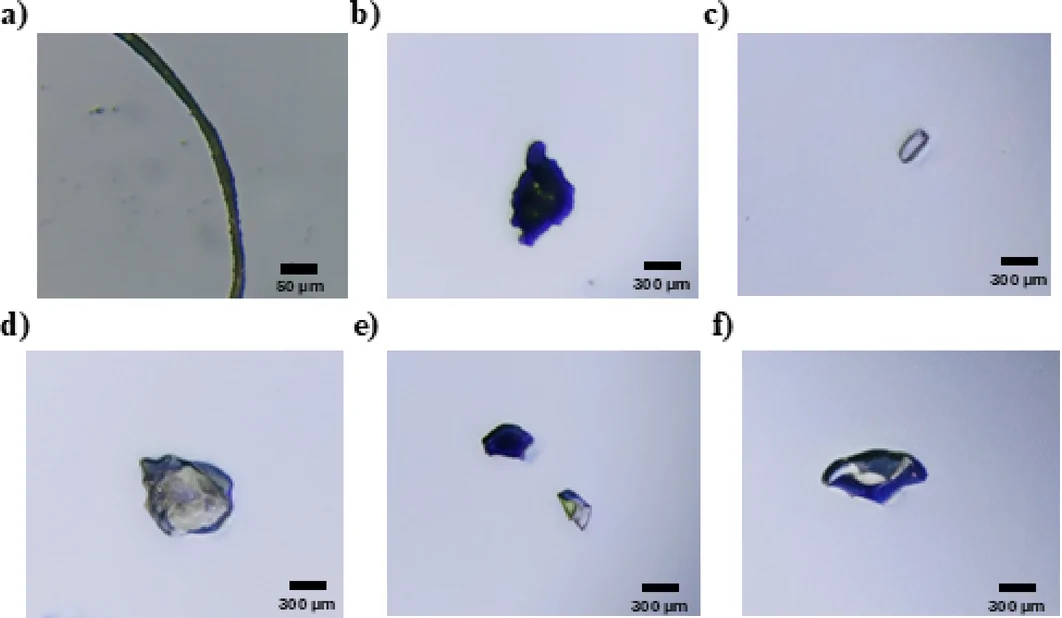
Visually identified images of (**a**) nylon fibre, (**b**) car tyre fragment, (**c**) PE granule (**d**) PP fragment, (**e**) fragments of car tyre and PE, (**f**) PE fragment.

Taking the above into account, ATR–FTIR analysis was used as a complementary technique to confirm the presence and general types of microplastic particles, rather than for quantitative assessment. Comparative analysis of the ATR-FIR spectra registered for the residue obtained after evaporation of hexane solvent from previously prepared extracts with the OPUS (Bruker) database of spectral indicated the presence of polymer types associated with Tire (Rubbers), Nylon 6 (polyamides, PA), LDPE (polyethylenes), Epcar 5875 (polypropylenes), PVA (polyvinyl alcohol), PP (polypropylenes). What is more important, the recorded spectra did not show the presence of bands corresponding to hexane, which indicates complete evaporation of the solvent from the samples. Figure 2a–f presents representative microscopic images of various shapes of MPs found in spider web samples. These shapes were identified as fibers, granules, and fragments, which are consistent with secondary microplastics formed through fragmentation of larger plastic materials. We were able to observe black, white, and gray or transparent microparticles. The particle sizes varied between 300 and 500 μm. The combined evidence from GC–MS (rubber-associated compounds) and ATR–FTIR (solid particle spectral identification) suggests that the detected rubber-related material likely originated from tire wear particles (TWPs) generated by friction between tires and road surfaces, a major and well-documented source of urban airborne MPs^65^.

The distribution of individual MPs in tested samples is presented in Table 1. The assignment was made based on the comparison of the experimental spectra with known plastic, as shown in Figures S4-S18 in the Supplementary Materials.

The vast majority of obtained IR spectra contain the MP’s polymer signature directly overlaid on the protein signature of the spider silk. At the same time, as previously indicated, the GC-MS analysis revealed the presence of plastic additives at significantly high levels.

This overlap between the polymer signature and the spider silk protein spectrum constitutes strong evidence for a close physical association between MP particles and the silk matrix, indicating that particles are likely adhering to or embedded within the web structure.

This close physical association between MPs and silk fibers suggests the possibility of further physicochemical interactions occurring on the web surface.

MPs can act not only as passive collectors of airborne particles but also as active surfaces capable of adsorbing other environmental pollutants, such as polycyclic aromatic hydrocarbons (PAHs), heavy metals, and persistent organic pollutants (POPs)^68^.

On the other hand, additives and plasticizers present in the polymer structure can migrate from MPs to other substances deposited on the web, affecting the chemical composition of extracts and potentially interfering with analytical detection. Such interactions can lead to both overestimation and underestimation of some compounds during GC–MS and FTIR analyses, depending on their volatility and affinity for the polymer surface^30^.

An additional limitation of the method is the difficulty in detecting transparent or very fine (< 10 μm) particles, which may be invisible under microscopic observation or difficult to identify spectroscopically^18,20^. These factors emphasize the need for further refinement of sample preparation procedures and detection methods to obtain a more accurate characterization of MPs and co-occurring contaminants on spider webs.

**Table 1:** Summary of chemical compounds and microplastic types identified in spider web samples, including size classifications, detection frequencies, and location-based distribution.

Polymer	Size Range (µm)	Frequency	Urban	Suburban	Samples with presence
Tire (Rubbers)	< 100–500	100%	Yes	Yes	1, 2, 3, 4, 5, 6, 7, 8, 9, 10, 12, 13, 14, 15
Nylon 6	100–1000	40%	Yes	Yes	1, 2, 8, 10, 12, 13
LDPE	200–1500	13%	Yes	No	5, 11, 13
PP	200–1000	7%	Yes	No	11
PVA	100–500	20%	Yes	Yes	7, 15
EPCAR 5875	100–700	20%	Yes	Yes	11

### Polymer types, size classes and implications for exposure

The major group of compounds detected based on ATR–FTIR analysis was associated with tire-derived materials (rubber) (90%), and such compounds were also observed in all extracts by GC–MS. Nylon 6 was the second most frequently indicated polymer type (40 in the analyzed spider web samples). This product is used in automobile parts, plasticizers for concrete patching cement, and wire and cable insulation. It can be applied in the manufacture of rugs, carpets, textiles, clothing, and tire cords^69^. Epcar 5875 and PVA were detected as polymer-associated signals in 20% of the studied samples. Ethylene Propylene Diene Terpolymer (Epcar 5875) is used for the production of rubbers, in particular for door seals, window seals, trunk seals, hood seals, or wiper blades in vehicles^70^. In turn, polyvinyl alcohol is a polymer soluble in water used commercially as a protective film in laundry and dish detergent pods or as a sizing and finishing agent in the textile industry^71^. LDPE and PP were indicated in 13% and 7% of the studied samples, respectively. This may be related to the detection of associated compounds in all hexane extracts by GC–MS. In Table 1, MP types, estimated size categories, detection frequencies, distribution in urban and suburban locations, as well as their presence in individual samples are presented.

While the current GC–MS and ATR–FTIR methods used in our study do not allow for direct measurement of particle size, the size ranges presented in Table 1 were estimated based on published data describing typical dimensions of airborne MPs associated with tire wear particles, textile fibers, and common packaging-related polymers reported in the literature^8,65^.

These estimates provide context for potential environmental and health relevance, particularly for particles within the inhalable size range (< 500 μm). The most common and ubiquitous type of MP was rubber-associated material linked to road traffic. The diversity of MP types and sizes indicates multiple emission sources and potential health risks from exposure to fine fractions of inhalable MPs. The majority of spider webs contained compounds associated with common packaging-related polymers (e.g., PE, PP, PET, PS). In general, research shows that plastics used in packaging, such as PS, PE, PET, break down and produce MPs more quickly in the air than in water^72^. According to Enyoh et al^8^., the most common polymers in the air include PS, PET, PVC, polyurethane (PUR), polyacrylonitrile (PAN), PA, PE, and PP. Numerous studies indicate that vehicle tire abrasion is a major source of MPs in urban air^65^, which is consistent with our observations in Wrocław.

This is the first time that spider webs have been applied for MPs assessment in Poland. Webs were used as passive natural samplers, making them suitable for studies on anthropogenic contamination. Inorganic components such as lead, zinc, nickel, copper, and ferromagnetic particles have previously been studied in spider webs^11^, whereas studies on MPs in spider webs remain limited (Table 2). Previous studies of that type have reported different dominant polymer types depending on location, including PU and PET in Brazil^73^, PET, PVC, and tire-derived materials in Germany^12^, and PE in Sweden^5^. These differences likely reflect regional variability in emission sources, whereas in the present study, tire-derived materials and polyamide-related compounds were most prominent.

Comparative data from airborne MP deposition studies in Poland and Central Europe remain scarce.

Within Poland, Szewc et al^74^. reported atmospheric MP deposition fluxes of 0–30 MPs m⁻² day⁻¹at a coastal urban site in Gdynia, with fibres as the dominant morphotype. Jarosz et al^75^. characterized MP in atmospheric deposition in Kraków across seven seasonal sampling cycles. Using ATR–FTIR and Py-GC/MS, the authors identified LDPE, Nylon-66, PET, and PP as prevailing polymers, broadly consistent with our findings despite methodological differences between atmospheric fallout collection and spider-web passive sampling. To our knowledge, no spider-web-based study of airborne MPs has been conducted in Lower Silesia or the Wrocław region prior to this work. At the broader Central European scale, the studies most directly comparable in terms of passive sampling matrix are those of Goßmann et al^12^. and Iordachescu et al^5^., also using spider webs, as discussed above. The predominance of tire-derived particles in our dataset, contrasting with PE dominance in Uppsala^5^ and PET/PVC in Oldenburg^12^, is likely attributable to the specific emission profile of Wrocław as one of the most traffic-congested cities in Central Europe. However, methodological heterogeneity across studies, including differences in sampling duration, preparation protocols, and analytical resolution, precludes robust quantitative comparison. This highlights the need for more standardized passive monitoring frameworks for airborne MPs.According to a global traffic ranking, Wrocław is among the most congested cities in its population category, with drivers spending significant time in traffic annually (https://www.tomtom.com/traffic-index/ranking/). This may explain the predominance of traffic-related MPs observed in this study^64^.

Although direct comparison with active air sampling methods was beyond the scope of this study, the types of MPs identified correspond to those reported in studies using conventional aerosol sampling^12,21^. Together, these observations highlight the potential of spider webs for qualitative airborne MP assessment. Previous studies (e.g^12,24^). have shown that spider webs can effectively capture airborne particles, including MPs, in a spatially resolved and cost-effective manner. Advantages include passive accumulation, lack of energy requirements, and applicability in infrastructure-limited environments. However, limitations include dependence on spider behavior, lower quantitative precision compared to active samplers, and sensitivity to environmental degradation. Therefore, spider webs should be considered complementary tools rather than replacements for standardized sampling methods.

Our study is not intended to replace standard methods but to complement them with a low-cost, maintenance-free approach, particularly useful in areas where active sampling is not feasible. Additionally, spider webs may preferentially capture lighter airborne particles, and therefore the results may be biased toward the inhalable fraction of MPs, which is particularly relevant from a human exposure perspective.

The approach proposed in this study has potential global applicability due to the widespread occurrence of Agelenidae spiders across different climatic zones, allowing for standardized passive monitoring of airborne MPs in diverse environments.

**Table 2:** Dominant polymers recorded on spider webs in previous studies.

Polymer type	Site	References
PE- dominant	Uppsala, Sweden	^5^
PU, PET- dominant	The Federal University of Espírito Santo (UFES) in southeastern Brazil	^73^
PET, PVC, CTT (car tire tread) and TTT (truck/bus tire tread.	Oldenburg, Germany	^12^
car tire tread CTT	Wrocław, Poland	this study

## Conclusions

This study provides the evidence that spider webs can serve as sensitive, naturally occurring biosamplers of airborne MPs in Poland. Due to their widespread occurrence and high density in urban environments, this method is particularly useful for identifying areas of increased concentration (“hotspots”) and supporting measurement planning using standard active techniques. Our findings show that webs consistently accumulate tire-derived particles and multiple polymer types across the urban landscape, revealing a surprisingly uniform pollution signal in a city with one of Europe’s highest traffic densities. However, spider webs are currently not widely used in studies on contamination by MPs, so it is crucial to develop this type of research in the future.

Currently, there are only a several studies devoted to the use of spider webs for MPs in air, none conducted in Poland; therefore, we think that our study’s findings offer a contribution to how spider webs might be used in similar investigations in the future. Although, the novelty of this work lies not only in the geographical context, but also in the combined use of spider webs as passive samplers with GC–MS and ATR-FTIR techniques for the analysis of leachable compounds and solid MPs in real-world urban conditions. We demonstrated that spider-web sampling with GC–MS and ATR-FTIR creates a dual analytical framework capable of capturing both chemical additives and solid polymer structures—a possibility rarely achieved in MP monitoring. The study also provides baseline data for a region with high traffic density, highlighting the presence of MPs within the inhalable fraction relevant to human exposure. In general, the absence of standardized data and procedures among the scientific community is one of the main constraints of research on MP pollution. We acknowledge that web sampling, although promising, requires further validation and standardization of methods (e.g., exposure time, sampling height, replicates) and cross-validation with active sampling methods. Despite these limitations, our study offers an accessible tool for initial surveillance of MP and opens the door to more structured multi-season, multi-method comparisons. It is an initial attempt to identify the quantity and type of airborne MPs in spider webs in a densely populated urban area, such as Wrocław in Poland.

## Supplementary Information

Below is the link to the electronic supplementary material.

Supplementary Material 1

## Data Availability

All data generated or analysed during this study are included in this published article and its Supplementary Information files.
