# Supplementary material for: Integrated GC–MS and ATR–FTIR analysis of airborne microplastics captured by spider webs
Source: Sci Rep. 2026 Jun 11;16:26630. doi: 10.1038/s41598-026-57675-z (PMC13503901; doi:10.1038/s41598-026-57675-z)
Supplement: Supplementary file 1 [file 41598_2026_57675_MOESM1_ESM.docx]

**Integrated GC–MS and ATR–FTIR Analysis of Airborne Microplastics Captured by Spider Webs.**

Supplementary materials

## Figure S1.

**Analytical workflow of spider-web sampling, sample preparation, GC–MS and ATR–FTIR analyses used for airborne microplastic assessment.**

**Table S1.** Description of sampling sites.

| **No.** | **Coordinates** | **Species** | **Description** |
| --- | --- | --- | --- |
| 1 | 51°06'44.4"N 17°03'25.8"E | *A. labyrinthica* | Curie Skłodowska Street, surrounded by old tenement houses and heavy vehicle and pedestrian traffic, next to the university, the webs were collected directly from the walls of tenement houses, traffic intensity during rush hours is about 600-700 cars per hour. |
| 2 | 51°06'34.7"N 17°03'14.6"E | *A. labyrinthica* | A small park, a tree-lined square at the Grunwaldzki Bridge, a suspension bridge over the Odra River, the central part of the city of Wrocław, the spider webs were collected directly from the vegetation, vehicle traffic intensity is about 800 cars per hour. |
| 3 | 51°06'34.4"N 17°03'10.8"E | *A. labyrinthica* | Sampling site situated directly on the Grunwaldzki Bridge over the Odra River. Any vegetation does not cover the area; the spider webs were collected directly from the bridge spans, there is a lot of pedestrian and car traffic on the bridge (about 800 cars per hour). |
| 4 | 51°06'34.0"N 17°03'08.1"E | *A. labyrinthica* | Another sampling site situated on the opposite side from the previous point, on the Grunwaldzki Bridge over the Odra River. Any vegetation does not cover the area; the webs were collected directly from the bridge spans, there is a lot of pedestrian and car traffic on the bridge (about 800 cars per hour). |
| 5 | 51°06'36.1"N 17°03'27.5"E | *A. labyrinthica* | Kazimierz Idaszewski square next to the building of the Wrocław University of Science and Technology, the spider webs were collected directly from the vegetation in the square, traffic intensity 800 cars per hour. |
| 6 | 51°06'36.0"N 17°03'27.6"E | *A. labyrinthica* | Kazimierz Idaszewski square next to the building of the Wrocław University of Science and Technology, the second sampling point, the spider webs were collected directly from the vegetation in the square, traffic intensity 800 cars per hour. |
| 7 | 51°06'36.5"N 17°03'23.6"E | *A. labyrinthica* | The vegetation surrounding the multi-lane road towards the Grunwaldzki Bridge, the webs were collected directly from the shrubs (traffic intensity approximately 800 cars per hour). |
| 8 | 51°06'36.3"N 17°03'23.4"E | *A. labyrinthica* | The vegetation surrounding the multi-lane road towards the Grunwaldzki Bridge, the second sampling point the webs were collected directly from the shrubs (traffic intensity approximately 800 cars per hour). |
| 9 | 51°06'36.2"N 17°03'23.2"E | *A. labyrinthica* | The vegetation surrounding the multi-lane road towards the Grunwaldzki Bridge, the third sampling point near the building of the Wrocław University of Science and Technology, samples were taken directly from the walls of the building (traffic intensity approximately 800 cars per hour). |
| 10 | 51°06'36.8"N 17°03'23.7"E | *A. labyrinthica* | The vegetation surrounding the multi-lane road towards the Grunwaldzki Bridge, the fourth sampling point the webs were collected directly from the shrubs (traffic intensity approximately 800 cars per hour). |
| 11 | 51°06'07.2"N 17°05'41.8"E | *A. labyrinthica* | Siemiradzkiego Street, in the eastern part of Wrocław, nearby single-family buildings and small tenement houses, home gardens, samples were taken directly from the vegetation in the garden, traffic intensity several cars per hour. |
| 12 | 51°06'07.0"N 17°05'41.9"E | *A. labyrinthica* | Siemiradzkiego Street, in the eastern part of Wrocław, nearby single-family buildings and small tenement houses, home gardens, the second sampling point, samples were taken directly from the vegetation in the garden, traffic intensity several cars per hour. |
| 13 | 51°06'07.3"N 17°05'41.6"E | *A. labyrinthica* | Siemiradzkiego Street, in the eastern part of Wrocław, nearby single-family buildings and small tenement houses, home gardens, the third sampling point, samples were taken directly from the vegetation in the garden, traffic intensity several cars per hour. |
| 14 | 51°05'59.1"N 17°05'37.4"E | *T. ferruginea* | Siemiradzkiego Street at the intersection with Chemońskiego Street, a street with more pedestrian and street traffic, single houses and villas in the area, the spider webs were taken from the wall surrounding the house, traffic intensity 50 cars per hour. |
| 15 | 51°05'59.3"N 17°05'37.6"E | *T. ferruginea* | Siemiradzkiego Street at the intersection with Chemońskiego Street, a street with more pedestrian and street traffic, single houses and villas in the area, the second sampling point, the spider webs were taken from the wall surrounding the house, traffic intensity 50 cars per hour. |

**Table S2.** Mass of analyzed spider web samples.

| Sample name | Sample mass [mg] |
| --- | --- |
| 1 | 26.2 |
| 2 | 8.9 |
| 3 | 15.5 |
| 4 | 4.4 |
| 5 | 8.4 |
| 6 | 9.9 |
| 7 | 11.6 |
| 8 | 3.0 |
| 9 | 9.9 |
| 10 | 2.6 |
| 11 | 1.5 |
| 12 | 2.4 |
| 13 | 3.1 |
| 14 | 32.8 |
| 15 | 4.5 |

**Figure S2.** Comparison of GC–MS chromatograms of spider webs following extraction with n-hexane (the numerical designations correspond to the ordinal number of the compounds presented in Table S3).

**Table S3.** Chemical compounds derived from plastic and identified in hexane extracts of spider web samples by GC–MS.

| **Ref.** | Suchocki *et al* 2023 | | Sait 2019 | Emecheta 2024 | Sarker and Rashid 2013 | Tran T M *et al* 2015,, Cederberg and Jensen 2017 | Williams 2013, National Toxicology Program 2019 | | | | Mohan *et al* 2021, | Williams 2013, National Toxicology Program 2019 | Cederberg and Jensen 2017 | Xu *et al* 2019, Okpashi *et al* 2020 | Emecheta 2024 | Rani M *et al* 2015, Ibarra *et al* 2019, Völker *et al* 2022, Cabanes *et al* 2020 | Sarker 2011, Al-Areqi *et al* 2021, Petrulionienė *et al* 2023 | Cabanes *et al* 2020, | Cederberg and Jensen 2017 | Chapke *et al* 2022 | Meboso *et al* 2023, Emecheta 2024 | Sait 2019 | Cabanes *et al* 2020, Özçakır and Karaduman 2020 | Völker *et al* 2022, Zhang T *et al* 2024 | Keresztes *et al* 2013, Erythropel *et al* 2014, Li *et al* 2022 | Ibarra *et al* 2019, Völker *et al* 2022, Chapke *et al* 2022 | Hill *et al* 2018, NCBI 2025 | Taghavi *et al* 2021, Abedeen *et al* 2021, | Ibarra *et al* 2019, Chapke *et al* 2022 | Völker *et al* 2022 | Völker *et al* 2022 | Taghavi *et al* 2021, Abraham *et al* 2017 |
| --- | --- | --- | --- | --- | --- | --- | --- | --- | --- | --- | --- | --- | --- | --- | --- | --- | --- | --- | --- | --- | --- | --- | --- | --- | --- | --- | --- | --- | --- | --- | --- | --- |
| **Function of compound** | Constituents of rubber | | VOCs solvent/fragnance | Chemical additive - plasticizer | Constituents of rubber | Constituents of silicones | Constituents of rubber | Constituents of rubber | Constituents of rubber | Constituents of rubber | Constituents of rubber | Constituents of rubber | Constituents of silicones | Chemical additive - plasticizer | Chemical additive - antioxidant | Chemical additive - slip agent | Constituents of PP | VOCs emitted from PE | Constituents of silicones  camethyl- | Chemical additive - lubricant | Chemical additive - slip agent | Chemical additive - lubricant | Constituents of PE | Biological degradation product | Chemical additive - plasticizer | Chemical additive - slip agent | Chemical additive - lubricant  hydroxypropyl ester | Biological degradation product | Chemical additive - slip agent, | Chemical additive - plasticizer | Chemical additive - plasticizer | Biological degradation product |
| **Polymer** | PP | PAN | | PUR | Rubber | Silicone | Rubber | Rubber | Rubber | Rubber | Rubber | Rubber | Silicone | PET | Rubber | PE | PP | PE | Silicone | PE | PA | PAN | PE | PS | PET | PE | Rubber | Unidentified | PE | PCV | PCV | Unidentified |
| **RT(min)** | 6.36 | | 6.41 | 8.01 | 8.17 | 8.53 | 9.26 | 9.82 | 10.13 | 10.57 | 10.72 | 12.26 | 12.60 | 13.06 | 13.47 | 13.59 | 13.65 | 14.41 | 14.61 | 14.74 | 14.94 | 15.05 | 16.78 | 16.91 | 17.14 | 17.26 | 17.97 | 18.28 | 18.30 | 18.57 | 18.64 | 23.76 |
| **Compound** | Heptane,2,5,5-trimethyl- | | Nonanal | Nonanoic acid | Dodecane, 4,6-dimethyl- | Cyclohexasiloxane, dodecamethyl- | Tetradecane | Dodecane , 2,6,11 trimethyl-  Dodecane, 2,6,11-trimethyl- | Pentadecane | Hexadecane | Hpptadecane | Heptadecane, 3-methyl- | Heptasiloxane, hexadecamethyl- | Phthalic acid, butyl tetradecyl ester | 7,9-Di-tert-butyl-1-oxaspiro(4,5)deca-6,9-diene-2,8-dione | n-Hexadecanoic acid | Eicosane | n-Nonadecanol-1 | Cyclooctasiloxane, hexadecamethyl- | 2-methylhexacosane | 9-Octadecenamide, (Z)- | Octadecanamide | Hexatriacontane | Hexadecanoic acid, 2-hydroxy-1-(hydroxymethyl)ethyl ester | Bis(2-ethylhexyl) phthalate (DEHP) | cis-11-Eicosenamide | Octadecanoic acid, 2,3-dihydroxypropyl ester | Tetrapentacontane | 13-Docosenamide, (Z)- | Phthalic acid, bis(7-methyloctyl) ester | 1,2-Benzenedicarboxylic acid, dinonyl ester | 1,4-Epoxynaphthalene-1(2H)-methanol,4,5,7-tris(1,1-dimethylethyl)-3,4-dihydro  - |
| **Lp** | **1** | | **2** | **3** | **4** | **5** | **6** | **7** | **8** | **9** | **10** | **11** | **12** | **13** | **14** | **15** | **16** | **17** | **18** | **19** | **20** | **21** | **22** | **23** | **24** | **25** | **26** | **27** | **28** | **29** | **30** | **31** |

**Figure S3.** The correlation coefficient matrix.

**Table S4.** Detailed results of the GC-MS analysis (area of signals 1-31 for samples 1-15 were determined based on TIC (total ion chromatogram)).

| **15** | 8764 | 151221 | 14370 | 11012 | 99321 | 278691 | 10044 | 47015 | 20845 | 35237 | 25991 | 48549 | 2760 | 36030 | 54382 | 69225 | 10753 | 41235 | 29874 | 37289 | 315680 | 77812 | 1349 | 43470 | 7719 | 156464 | 104772 | 0 | 21476 | 13436 | 70590 |
| --- | --- | --- | --- | --- | --- | --- | --- | --- | --- | --- | --- | --- | --- | --- | --- | --- | --- | --- | --- | --- | --- | --- | --- | --- | --- | --- | --- | --- | --- | --- | --- |
| **14** | 10249 | 155348 | 54235 | 11617 | 153907 | 331559 | 7730 | 52292 | 22521 | 44847 | 32934 | 64963 | 46295 | 120670 | 405995 | 111138 | 130745 | 68171 | 213330 | 151330 | 874420 | 1129554 | 0 | 0 | 87067 | 156478 | 0 | 595701 | 0 | 11270 | 0 |
| **13** | 30299 | 44847 | 17460 | 62055 | 159832 | 339405 | 51480 | 160300 | 33448 | 65647 | 32610 | 80810 | 6744 | 76651 | 27788 | 136087 | 4760 | 65193 | 62560 | 39193 | 44795 | 44321 | 141404 | 41762 | 16770 | 61708 | 62560 | 136666 | 141404 | 41762 | 61708 |
| **12** | 28949 | 40812 | 21507 | 49105 | 43145 | 241215 | 37986 | 130105 | 32006 | 46834 | 58329 | 47414 | 22398 | 122758 | 69100 | 179410 | 8264 | 38269 | 114831 | 10070 | 8013 | 830622 | 42871 | 103275 | 0 | 220315 | 2959968 | 0 | 391170 | 797717 | 269516 |
| **11** | 2112 | 31267 | 0 | 28510 | 57579 | 263075 | 41021 | 77121 | 12682 | 26317 | 28282 | 41150 | 4694 | 61154 | 33620 | 60921 | 11518 | 31433 | 16690 | 14502 | 117391 | 37871 | 1178591 | 31339 | 17716 | 358982 | 11920 | 0 | 0 | 8874 | 200078 |
| **10** | 18957 | 133020 | 28019 | 24168 | 56446 | 312593 | 7281 | 62979 | 28106 | 39565 | 35146 | 49007 | 11477 | 29322 | 18439 | 92366 | 8888 | 44927 | 29451 | 17712 | 185621 | 31700 | 0 | 54281 | 17295 | 9734 | 0 | 199570 | 0 | 63706 | 88526 |
| **9** | 22138 | 35412 | 3421 | 40902 | 83448 | 238113 | 36549 | 99382 | 24227 | 39875 | 22335 | 58287 | 7160 | 77980 | 55825 | 99331 | 5801 | 57817 | 137163 | 6817 | 55396 | 196773 | 109302 | 45624 | 10885 | 99770 | 113240 | 0 | 32787 | 15521 | 120447 |
| **8** | 1104 | 195023 | 28147 | 8288 | 96641 | 193637 | 3207 | 15576 | 10005 | 15452 | 13810 | 39209 | 118608 | 49188 | 36707 | 46456 | 58443 | 46035 | 12561 | 333537 | 1686919 | 49431 | 18270 | 471291 | 62875 | 50883 | 90581 | 0 | 0 | 9949 | 94104 |
| **7** | 18448 | 36865 | 0 | 48936 | 171320 | 388963 | 70890 | 171617 | 27834 | 67372 | 39130 | 75999 | 9902 | 113433 | 60960 | 137374 | 9378 | 62887 | 83542 | 15091 | 55522 | 112246 | 218007 | 66454 | 2617 | 113132 | 107967 | 0 | 0 | 9666 | 327737 |
| **6** | 26039 | 35265 | 30510 | 46126 | 75748 | 241997 | 24631 | 110961 | 33253 | 40954 | 35347 | 57659 | 5046 | 50620 | 62653 | 136713 | 9523 | 54958 | 70064 | 6492 | 36408 | 56738 | 88977 | 82233 | 0 | 60871 | 133398 | 0 | 24702 | 31041 | 155426 |
| **5** | 18380 | 57122 | 38875 | 73319 | 80497 | 500675 | 107655 | 276165 | 36868 | 92036 | 61790 | 48992 | 20979 | 173769 | 77591 | 161703 | 6359 | 33055 | 165519 | 29972 | 326379 | 93298 | 2631142 | 75453 | 30670 | 1073906 | 52127 | 0 | 0 | 14493 | 645218 |
| **4** | 8025 | 126675 | 19715 | 13601 | 91200 | 302460 | 3976 | 48538 | 23083 | 43927 | 35764 | 50998 | 6378 | 38142 | 27006 | 83451 | 7181 | 49749 | 20436 | 41777 | 281864 | 59652 | 3721 | 43645 | 14812 | 45534 | 90038 | 0 | 0 | 0 | 53386 |
| **3** | 15164 | 37874 | 63359 | 46319 | 106840 | 352080 | 57266 | 161636 | 27480 | 61553 | 36051 | 59400 | 19639 | 133142 | 92900 | 132877 | 12026 | 66330 | 116205 | 14328 | 134595 | 197594 | 634145 | 114315 | 6397 | 257893 | 241471 | 0 | 0 | 6614 | 290231 |
| **2** | 8247 | 130893 | 39266 | 12943 | 58858 | 343974 | 3000 | 49844 | 25284 | 46444 | 37740 | 46928 | 8124 | 46851 | 46044 | 79001 | 7738 | 35950 | 36914 | 45915 | 418573 | 118117 | 5792 | 66537 | 15393 | 80211 | 168658 | 0 | 0 | 6218 | 42778 |
| **1** | 3311 | 34984 | 38168 | 24474 | 204920 | 256056 | 38632 | 74954 | 12249 | 35525 | 18992 | 79274 | 150488 | 94890 | 146397 | 104962 | 52105 | 81128 | 96184 | 29347 | 149840 | 433225 | 1117869 | 484694 | 15267 | 471063 | 196089 | 0 | 0 | 12495 | 233936 |
| **RT(min)** | 6.36 | 6.41 | 8.01 | 8.17 | 8.53 | 9.26 | 9.82 | 10.13 | 10.57 | 10.72 | 12.26 | 12.60 | 13.06 | 13.47 | 13.59 | 13.65 | 14.41 | 14.61 | 14.74 | 14.94 | 15.05 | 16.78 | 16.91 | 17.14 | 17.26 | 17.97 | 18.28 | 18.30 | 18.57 | 18.64 | 23.76 |
| **CAS** | 1189-99-7 | 124-19-6 | 112-05-0 | 61141-72-8 | 540-97-6 | 629-59-4 | 629-78-7 | 112-95-8 | 544-76-3 | 629-94-7 | 6418-44-6 | 541-01-5 | 256481-49-9 | 82304-66-3 | 57-10-3 | 646-31-1 | 1454-84-8 | 556-68-3 | 1561-02-0 | 301-02-0 | 124-26-5 | 630-06-8 | 23470-00-0 | 117-81-7 | 10436-08-5 | 123-94-4 | 5856-66-6 | 112-84-5 | 20548-62-3 | 84-76-4 | 56771-86-9 |
| **Lp** | **1** | **2** | **3** | **4** | **5** | **6** | **7** | **8** | **9** | **10** | **11** | **12** | **13** | **14** | **15** | **16** | **17** | **18** | **19** | **20** | **21** | **22** | **23** | **24** | **25** | **26** | **27** | **28** | **29** | **30** | **31** |

The figures present below show the comparison of the experimental spectra with the spectra of matching polymers. The individual color of the spectrum is assigned the following designations:

red line - sample spectrum

black line – car tire

green line - nylon 6

pink line - epcar 5875

teal line - polyethylene

purple line - polyvinyl alcohol

cyan line - polypropylene

**Figure S4.** Comparison of the ATR-FTIR spectrum registered for the residue obtained after evaporation hexane solvent from sample 1, with the spectrum of plastic fitted by OPUS (Bruker) database.

**Figure S5.** Comparison of the ATR-FTIR spectrum registered for the residue obtained after evaporation hexane solvent from sample 2, with the spectrum of plastic fitted by OPUS (Bruker) database.

**Figure S6.** Comparison of the ATR-FTIR spectrum registered for the residue obtained after evaporation hexane solvent from sample 3, with the spectrum of plastic fitted by OPUS (Bruker) database.

**Figure S7.** Comparison of the ATR-FTIR spectrum registered for the residue obtained after evaporation hexane solvent from sample 4, with the spectrum of plastic fitted by OPUS (Bruker) database.

**Figure S8.** Comparison of the ATR-FTIR spectrum registered for the residue obtained after evaporation hexane solvent from sample 5, with the spectrum of plastic fitted by OPUS (Bruker) database.

**Figure S9.** Comparison of the ATR-FTIR spectrum registered for the residue obtained after evaporation hexane solvent from sample 6, with the spectrum of plastic fitted by OPUS (Bruker) database.

**Figure S10.** Comparison of the ATR-FTIR spectrum registered for the residue obtained after evaporation hexane solvent from sample 7, with the spectrum of plastic fitted by OPUS (Bruker) database.

**Figure S11.** Comparison of the ATR-FTIR spectrum registered for the residue obtained after evaporation hexane solvent from sample 8, with the spectrum of plastic fitted by OPUS (Bruker) database.

**Figure S12.** Comparison of the ATR-FTIR spectrum registered for the residue obtained after evaporation hexane solvent from sample 9, with the spectrum of plastic fitted by OPUS (Bruker) database.

**Figure S13.** Comparison of the ATR-FTIR spectrum registered for the residue obtained after evaporation hexane solvent from sample 10, with the spectrum of plastic fitted by OPUS (Bruker) database.

**Fig. S14.** Comparison of the ATR-FTIR spectrum registered for the residue obtained after evaporation hexane solvent from sample 11, with the spectrum of plastic fitted by OPUS (Bruker) database.

**Figure S15.** Comparison of the ATR-FTIR spectrum registered for the residue obtained after evaporation hexane solvent from sample 12, with the spectrum of plastic fitted by OPUS (Bruker) database.

**Figure S16a.** Comparison of the ATR-FTIR spectrum registered for the residue obtained after evaporation hexane solvent from sample 13, with the spectrum of plastic fitted by OPUS (Bruker) database.

**Figure S16b.** Comparison of the ATR-FTIR spectrum registered for the microplastic particle obtained after evaporation hexane solvent from sample 13, with the spectrum of plastic fitted by OPUS (Bruker) database.

**Figure S17.** Comparison of the ATR-FTIR spectrum registered for the residue obtained after evaporation hexane solvent from sample 14, with the spectrum of plastic fitted by OPUS (Bruker) database.

**Figure S18.** Comparison of the ATR-FTIR spectrum registered for the residue obtained after evaporation hexane solvent from sample 59, with the spectrum of plastic fitted by OPUS (Bruker) database.
